# Inhibition of glycogen synthase kinase 3 by lithium, a mechanism in search of specificity

**DOI:** 10.3389/fnmol.2022.1028963

**Published:** 2022-11-24

**Authors:** Dipashree Chatterjee, Jean Martin Beaulieu

**Affiliations:** Department of Pharmacology and Toxicology, University of Toronto, Toronto, ON, Canada

**Keywords:** lithium, bipolar disorder, kinesin, CREB, glycogen synthase kinase 3, FXR1, collapsin response mediator protein 2

## Abstract

Inhibition of Glycogen synthase kinase 3 (GSK3) is a popular explanation for the effects of lithium ions on mood regulation in bipolar disorder and other mental illnesses, including major depression, cyclothymia, and schizophrenia. Contribution of GSK3 is supported by evidence obtained from animal and patient derived model systems. However, the two GSK3 enzymes, GSK3α and GSK3β, have more than 100 validated substrates. They are thus central hubs for major biological functions, such as dopamine-glutamate neurotransmission, synaptic plasticity (Hebbian and homeostatic), inflammation, circadian regulation, protein synthesis, metabolism, inflammation, and mitochondrial functions. The intricate contributions of GSK3 to several biological processes make it difficult to identify specific mechanisms of mood stabilization for therapeutic development. Identification of GSK3 substrates involved in lithium therapeutic action is thus critical. We provide an overview of GSK3 biological functions and substrates for which there is evidence for a contribution to lithium effects. A particular focus is given to four of these: the transcription factor cAMP response element-binding protein (CREB), the RNA-binding protein FXR1, kinesin subunits, and the cytoskeletal regulator CRMP2. An overview of how co-regulation of these substrates may result in shared outcomes is also presented. Better understanding of how inhibition of GSK3 contributes to the therapeutic effects of lithium should allow for identification of more specific targets for future drug development. It may also provide a framework for the understanding of how lithium effects overlap with those of other drugs such as ketamine and antipsychotics, which also inhibit brain GSK3.

## Introduction

Lithium is the most mysterious drug used in psychiatry. Chemically, lithium ions can affect several biochemical functions. But how they regulate mood in bipolar disorder and other conditions remains poorly understood. This is problematic since lithium can be both very effective as a therapy and ultimately toxic for patients when administered over a lifetime (Volkmann et al., 2020). Inhibition of enzymes from the glycogen synthase kinase 3 (GSK3) family is a popular explanation for lithium’s therapeutic effects (O’Brien and Klein, 2009; Freland and Beaulieu, 2012; Alda, 2015; Haggarty et al., 2021). However, both GSK3α and GSK3β are regulatory hubs for several biological processes. It is thus unclear how inhibition of GSK3 can provide a solid heuristic foundation for the development of better alternatives to lithium therapy (Doble and Woodgett, 2003; Kaidanovich-Beilin and Woodgett, 2011; Sutherland, 2011; Kaidanovich-Beilin et al., 2012). Further understanding of mechanisms by which lithium acts beyond GSK3 inhibition is thus necessary.

Here, we present an overview of lithium therapy and its association with GSK3 inhibition. Afterward, we cover biological processes that are affected by lithium and GSK3 in relation to bipolar disorder. Finally, we focus on specific GSK3 direct substrates that are presumably important for lithium’s therapeutic effects. In doing so, we considered four main questions: (1)Does the substrate contribute to biological functions that are affected by lithium treatment? (2) Is the activity or expression of the substrate affected by lithium and GSK3 inhibition? (3) Is the regulation of the substrate by lithium and GSK3 inhibition compatible with therapy? and (4) Is the effect of lithium on the substrate dependent upon a direct or indirect inhibition of GSK3?

## Lithium therapy

Lithium salts—of citrate or carbonate—are commonly prescribed as mood stabilizers to manage symptoms of bipolar disorder, a mental illness affecting up to 2.5% of the population (Merikangas et al., 2011; Iria et al., 2016). Bipolar disorder is characterized by a cycling of mood between depressive episodes and episodes of mania or hypomania (American Psychiatric Association and DSM-5 Task Force, 2013). Lithium maintenance therapy has been demonstrated to reduce the risk for both types of episodes in adult populations (Schou et al., 1954; Burgess et al., 2001;Volkmann et al., 2020). Lithium can also be effective as part of therapies to address depressive or manic/hypomanic symptoms in cyclothymia (Barroilhet and Ghaemi, 2020), major unipolar depression (De Montigny, 1994; Barroilhet and Ghaemi, 2020; Price et al., 2021), and schizophrenia (Leucht et al., 2015). Furthermore, lithium therapies have been shown to reduce suicidality (Kessing et al., 2005; Del Matto et al., 2020) and improve sleep (Billiard, 1987; Geoffroy et al., 2016).

Despite its demonstrated efficacy, lithium is not always a first-choice medication for treating bipolar disorder. Alternative pharmacotherapies mostly involve second generation antipsychotics and a handful of “mood stabilizing” anticonvulsant drugs (e.g., valproic acid, lamotrigine, and carmabazepine). However, it should be noted that in contrast to lithium, these pharmacological agents do not address the full spectrum of bipolar disorder symptoms and lack prophylactic action (Fountoulakis and Vieta, 2008; Zhihan et al., 2022). Hesitancy toward lithium may be explained by its various serious long-term side effects, narrow therapeutic window, and by a lack of clear mechanisms explaining its mood regulating actions (Volkmann et al., 2020). Uncovering how lithium affects mood to exert therapeutic action in bipolar disorder would not only improve clinical practice using lithium, but also allow for the development of more effective and safer therapeutic alternatives.

## Direct and indirect inhibition of GSK3 by lithium

Several mechanisms have been proposed over the years for the regulation of mood by lithium (for review see: Freland and Beaulieu, 2012; Alda, 2015; Haggarty et al., 2021). Inhibition of GSK3α and GSK3β is presently one of the best supported mechanisms explaining the neuro-psychiatric effects of lithium. Indeed, multiple investigation using pharmacological inhibitors or genetic inactivation of GSK3 (by homologous recombination or CRISPR/Cas9 somatic approaches) have replicated correlates of lithium treatments in animal models and patient derived systems (Beaulieu et al., 2009; O’Brien and Klein, 2009; Freland and Beaulieu, 2012; Paul et al., 2020; Haggarty et al., 2021).

**Figure 1:**
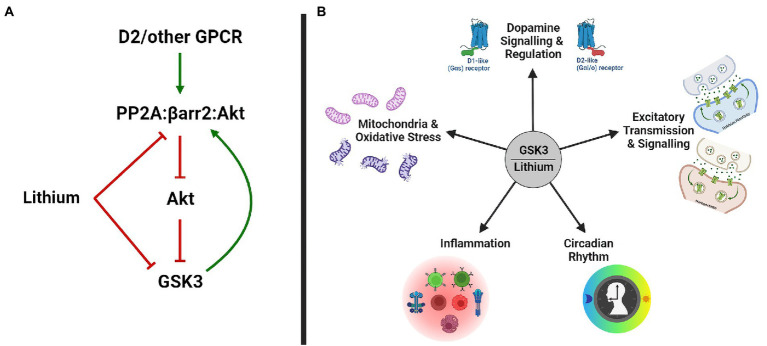
GSK3 and Lithium. **(A)** Lithium inhibits GSK3 directly and indirectly, by disrupting the PP2A/βarr2/Akt complex and increasing Akt activity. **(B)** Biological functions modulated by GSK3 and lithium in bipolar disorder. GSK3, glycogen synthase kinase-3; GPCR, g-protein coupled receptor; and PP2A, protein phosphatase 2A.

Direct inhibition of GSK3 by lithium (Figure 1A) has originally been uncovered in studies of development and differentiation in *Xenopus*, *Dictyostelium*, and *Drosophila* (Klein and Melton, 1996; Stambolic et al., 1996). This effect of lithium was later explained by a competition between Li^+^ and Mg^2+^ ions, preventing the association of Mg^2+^ to GSK3 kinases for which it is a co-factor (Ryves and Harwood, 2001; Sun et al., 2011). Lithium treatment resulting in therapeutic lithium serum levels (0.8–1.5 mM) also increases the inhibitory phosphorylation of GSK3 isoforms by Akt, thus providing an indirect mechanism (Figure 1A) for the inhibition of GSK3 by lithium (Chalecka-Franaszek and Chuang, 1999; Beaulieu et al., 2008). This indirect inhibition can be explained in part by an action of lithium ions on D2 dopamine receptor (DrD2) signaling. Activation of DrD2 results in the formation of a signaling complex in which Akt interacts with β-arrestin 2 and PP2A, ensuing in Akt dephosphorylation and inhibition (Beaulieu et al., 2004, 2005; O’Brien et al., 2011). Lithium has been shown to interfere with the interaction of Akt and β-arrestin 2, possibly *via* displacement of Mg^2+^ ions (Beaulieu et al., 2008). Disruption of the Akt/β-arrestin 2 signaling complex by lithium results in increased Akt activity and subsequent indirect inhibition of GSK3, therefore antagonizing the action of DrD2 on this pathway.

## Biological functions modulated by GSK3 and lithium

The two GSK3 kinases are ubiquitously expressed enzymes, which have partially overlapping functions (Kaidanovich-Beilin and Woodgett, 2011; Ahmad and Woodgett, 2020). Not surprisingly these kinases also have about 100 validated substrates (Kaidanovich-Beilin and Woodgett, 2011) and several more predicted ones (Taelman et al., 2010). Furthermore, the central role of GSK3 in myriads of biological functions keeps their genes under strong selective pressure. This probably explains why few rare and no common variants of *GSK3A* or *GSK3B* genes have been associated with enhanced risk for bipolar disorder (Benedetti et al., 2013; Stahl et al., 2019; Li et al., 2021; Mullins et al., 2021). Identification of GSK3 as a probable mediator of the effects of lithium is thus rather unsatisfactory, as it does not point toward a clear process by which it can stabilize mood. Attempts to better understand the contribution of GSK3 inhibition to lithium effects have focused mostly on biological functions that are affected both by GSK3 and lithium (Figure 1B). However, this is not a simple task since GSK3 activity is involved in the regulation of gene expression, cytoskeletal organization, metabolism, mRNA regulation, inflammation, and neurotransmission, to name a few (Beaulieu et al., 2009; Kaidanovich-Beilin and Woodgett, 2011; Freland and Beaulieu, 2012; Beurel and Jope, 2014; Beurel et al., 2015; Snitow et al., 2021).

### Dopamine signaling and regulation

Dopamine neurotransmission is suspected to play a role in the pathophysiology of bipolar disorder (van Enkhuizen et al., 2015). The dopamine hypothesis suggests a faulty homeostatic mechanism in which there is hyperdopaminergic tone in the manic phase, leading to overcompensation and a rapid decline into the depressive phase characterized by hypodopaminergic tone, which switches back to mania during a failed compensation (Tissot, 1975).

Some support for this hypothesis in humans comes from the efficacy of antipsychotic drugs in correcting manic symptoms of bipolar disorder (Gelenberg and Hopkins, 1996; Buoli et al., 2014). While, targeting several receptors, these drugs are all antagonists or partial agonists of DrD2 (Miyamoto et al., 2005). In schizophrenia, it has been demonstrated that clinical efficacy of antipsychotics is directly correlated with DrD2 antagonism, and long-term treatment results in diminished dopamine cell firing as well as decreased basal dopamine levels (Creese et al., 1976; Seeman et al., 1976; Amato et al., 2011). It is thus probable that antipsychotics also exert their effects on mania by regulating the efficacy of dopamine transmission. In addition, imaging studies point toward changes of dopamine homeostasis or receptor expression in the striatum and prefrontal cortex of people expressing different symptomatic phases of bipolar disorder (Berk et al., 2007; Ashok et al., 2017; Jauhar et al., 2017). Altered expression of dopamine receptors in post-mortem brains has also been associated with bipolar disorder (Zhan et al., 2011; Kaalund et al., 2014).

Animal models having elevated dopamine tone resulting from reduced expression of the dopamine transporter (DAT), or administration of pharmacological DAT inhibitors, replicate several motor and cognitive endophenotypes of bipolar disorder, which can be corrected by lithium and other mood stabilizers (Beaulieu et al., 2004; van Enkhuizen et al., 2015; Milienne-Petiot et al., 2017). Furthermore, pharmacological inhibition of GSK3 or inactivation of GSK3β, using recombinant alleles or intersectional somatic CRISPR/Cas9 approaches in neuronal populations expressing dopamine receptors, replicates behavioral outcomes of lithium treatment in mice (Beaulieu et al., 2004, 2009; Urs et al., 2012; Khlghatyan and Beaulieu, 2020).

Lithium has been shown to affect both dopamine release by neurons of the mesolimbic system and signaling response to dopamine downstream of its receptors (Beaulieu, 2016). Chronic administration of lithium has been shown to prevent excessive and maladaptive release of dopamine, without reducing basal dopamine tone (Can et al., 2016). The contribution of GSK3 inhibition to this effect has not been fully elucidated. However, it is notable that mice expressing a dominant negative GSK3β transgene display reduced DrD2 auto-receptor functions, and thus release more dopamine (Gomez-Sintes et al., 2014).

In neurons receiving dopamine innervation, acute lithium and chronic lithium have been shown to antagonize β-arrestin 2-mediated DrD2 signaling. Activation of DrD2 leads to an inhibition of Akt and concomitant relief of GSK3 inhibition. In contrast, antagonism of this pathway by lithium results in an indirect inhibition of GSK3, resulting from increased Akt activity (Beaulieu et al., 2004, 2005, 2008). Interestingly, mice lacking β-arrestin 2 expression displayed blunted responsiveness to lithium in behavioral tests having predict validity for anti-manic and antidepressant drug actions. This deficiency of responsiveness was correlated with an absence of indirect GSK3 inhibition both in β-arrestin 2 and DrD2 congenital knockout animals (Beaulieu et al., 2008; Del' Guidice and Beaulieu, 2015; Del'Guidice et al., 2015).

In addition to DrD2 signaling, lithium may also affect cyclic AMP driven signaling downstream of the D1 dopamine receptor (DrD1). Indeed, the transcription factor cAMP response element-binding protein (CREB) is a major target on which both DrD1 and GSK3 signaling can converge (Wang et al., 1994; Beaulieu and Gainetdinov, 2011; Rana et al., 2022). To our knowledge, the possibility of an interaction between DrD1 and GSK3 modulation of CREB activity has not been directly tested in the context of a contribution to the full scope of lithium effects in rodents. However, conditional knockout of GSK3β specifically in DrD1 expressing neurons of the striatum did not replicate inhibitory effects of lithium on amphetamine induced hyperactivity (Urs et al., 2012).

### Excitatory transmission and signaling

Increased glutamate transmission and loss of inhibitory regulation leading to an excitatory/inhibitory neurotransmission imbalance and excitotoxic insults have been reported in post-mortem studies of bipolar disorder (Hashimoto et al., 2007; Rao et al., 2010). Furthermore, altered expression of vesicular glutamate transporter 1 (vGLUT1) that could lead to increased quantal glutamate release have also been observed (Uezato et al., 2009; Eastwood and Harrison, 2010).

Increased glutamatergic tone in bipolar disorder could result from a dysregulation of both Hebbian [long-term potentiation (LTP) and long-term depression (LTD)] as well as non-Hebbian (homeostatic) plasticity, to which dysregulated GSK3 activity can contribute (Peineau et al., 2007; Bradley et al., 2012; Machado-Vieira, 2018; Tatavarty et al., 2020). Overall, activation of GSK3 promotes LTD, while its inhibition promotes LTP, although this is not always the case (Hooper et al., 2007; Peineau et al., 2007; Zhu et al., 2007). GSK3 is also important for the homeostatic regulation of glutamatergic transmission involved in cell autonomous synaptic scaling and system level sleep homeostasis (Khlghatyan et al., 2020). Moreover, lithium rescues SHANK3-deficiency-induced deficits of homeostatic synaptic scaling as a result of its inhibitory effect on GSK3 (Tatavarty et al., 2020).

Investigations in animals indicate that both acute and chronic lithium treatments affect glutamate synaptic concentration, glutamate reuptake, and ionotropic NMDA/AMPA receptor signaling, albeit with variable results across experimental systems (Dixon and Hokin, 1998; Nonaka et al., 1998; Du et al., 2004). Furthermore, chronic lithium treatment of cortical neurons shifts excitatory/inhibitory equilibrium of synaptic networks toward inhibition (Khayachi et al., 2021). This is supporting a model in which lithium reduces excitatory neurotransmission to correct an imbalance of excitatory/inhibitory neurotransmission in bipolar disorder. However, direct modulation of components of glutamate synapses by lithium and GSK3 are probably not the only contributing factors since lithium can also modulate neuronal morphology in response to allostatic pressure such as chronic stress (Wood et al., 2004; Gray and Mcewen, 2013).

### Circadian rhythm and sleep

Disruptions of circadian rhythm and sleep are established endophenotypes of bipolar disorder. Patients with bipolar disorder are sensitive to effects of sleep deprivation, have aberrant sleep–wake cycles, alterations in sleep patterns, REM sleep, and sleep duration that are accompanied by increased number of arousals and decreased sleep efficiency (Moody and Allsopp, 1969; Kripke et al., 1978; Mansour et al., 2005; Rock et al., 2014).

The primary circadian transcription factors (CLOCK and BMAL1) heterodimerize and control rhythmic expression of clock genes *Per* and *Cry*. CLOCK/BMAL1 increases levels of PER/CRY proteins, which then translocate to the nucleus to implement a negative feedback loop by repressing CLOCK/BMAL1 (Jin et al., 1999; Lee et al., 2001; Lowrey and Takahashi, 2004). Expression of a dominant negative variant of Clock (ClockΔ19) in mice results in a mix of manic and depressive like phenotypes that are reminiscent of bipolar disorder (Mcclung, 2007; Roybal et al., 2007; Mukherjee et al., 2010). Interestingly, ClockΔ19 mice display decreased AMPA-mediated excitatory synaptic responses and GluA1 AMPA subunit levels in the *nucleus accumbens*. Overexpressing GluA1 normalized mania-like behavior in these mice (Parekh et al., 2018). Furthermore, ClockΔ19 mice also display exacerbated striatal dopamine release, altered expression of dopamine receptors, and increased DrD2 to DrD1 ratio (Spencer et al., 2012).

Circadian activity is regulated by a master pacemaker located in the suprachiasmatic nucleus (SCN) of the hypothalamus (Weaver, 1998). GSK3β activity in the SCN oscillates over a 24-h period, where inhibition is highest during the night. Furthermore, GSK3β regulates the phosphorylation and stability of circadian proteins by regulating CLOCK/BMAL1 and PER/CRY (Iwahana et al., 2004; Paul et al., 2012; Jaworski, 2020). Accordingly, GSK3β haploinsufficiency affects circadian rhythm by lengthening circadian locomotor activity period, and inhibiting GSK3β mitigates ClockΔ19 mice hyperactivity (Kozikowski et al., 2011; Lavoie et al., 2013).

Lithium modulates clock gene expression, lengthens circadian period, and resynchronizes circadian rhythms (Hofmann et al., 1978; Johnsson et al., 1979; Mccarthy et al., 2012, 2013). In a paradoxical sleep deprivation model of mania, lithium was able to reverse behavioral alterations, and bipolar disorder patients that do not respond to lithium treatment have significantly increased activity of GSK3β and decreased expression of circadian clock genes (Geoffroy et al., 2018; Dal-Pont et al., 2019). Additionally, lithium rescued phase-signaling dysfunction in the *nucleus accumbens* of ClockΔ19 mice (Dzirasa et al., 2010).

### Inflammation

Increased inflammation has been implicated in multiple brain pathologies, including bipolar disorder, schizophrenia, major depression, and suicidal behavior (Nässberger and Träskman-Bendz, 1993; Dowlati et al., 2010; Miller et al., 2011; Munkholm et al., 2013; Beurel and Jope, 2014). In bipolar disorder, there are several increased inflammatory markers including tumor necrosis factor-α (TNFα), interleukin (IL)-1β, IL-6, IL-4, and IL-10 (Modabbernia et al., 2013). This inflammatory reaction can be modulated by GSK3. Activated GSK3 stimulates the production of pro-inflammatory cytokines (e.g., IL-6, IL-1β, and TNFα) and decreases the production of anti-inflammatory cytokines (e.g., IL-10; Martin et al., 2005; Huang et al., 2009; Jope et al., 2017). Whereas, inhibiting GSK3 reversed these effects, especially in microglia and astrocytes, in turn preventing inflammation-induced neural toxicity (Martin et al., 2005; Beurel and Jope, 2009; Yuskaitis and Jope, 2009). Moreover, GSK3 has been shown to promote inflammation in relation to aggression- and depressive-like behaviors in mice (Martin et al., 2005; Sengupta et al., 2007; Garcia et al., 2008; Beurel and Jope, 2014; Cheng et al., 2016, 2018; Pavlov et al., 2019).

Lithium exerts anti-inflammatory properties by reducing pro-inflammatory cytokines (TNFα, IL-1β, and IL-6) and increasing anti-inflammatory cytokines (IL-2 and IL-10). These effects have been correlated with diminished aggression, impulsivity, and depressive symptoms in both human and model systems (Kishter et al., 1985; Maes et al., 1999; Martin et al., 2005; Knijff et al., 2007; Beurel and Jope, 2014; Nassar and Azab, 2014). However, a study did show that TNFα was increased in lithium-treated bipolar disorder patients compared to non-medicated patients and healthy controls (Guloksuz et al., 2010).

### Mitochondria and oxidative stress

Several studies have found a relationship between mitochondrial dysfunction and bipolar disorder, where there is impaired energy production and higher levels of oxidative stress, which can lead to inflammation (Kato and Kato, 2000; Andreazza et al., 2010, 2013; Machado et al., 2016; Jeong et al., 2020). Additionally, in bipolar disorder, mitochondrial gene expression is reduced and accelerated telomere shortening is suggested to be highly influenced by oxidative stress damage (Konradi et al., 2004; Simon et al., 2006). This mitochondrial dysregulation has been supported to be influenced by GSK3 directly and indirectly. For example, GSK3 has been shown to affect mitochondrial motility *via* effects involving disrupted-in-schizophrenia 1 (DISC1) and microtubule stability, thus perturbing mitochondrial transport to the dendrites and axons *via* motor proteins (Lipina et al., 2011; Dolma et al., 2014; Ogawa et al., 2014).

In addition, there is a separate mitochondrial pool of GSK3 (mGSK3) that modulates the Krebs cycle and oxidative phosphorylation, to maintain homeostatic redox equilibrium (Hoshi et al., 1995, 1996; King et al., 2008). Dysregulation of mGSK3 can result in various disorders. Inactivating mGSK3 can revert mitochondrial pathology by promoting mitochondrial biogenesis and dynamics, as well as attenuating mitochondrial permeability and mitochondrial apoptosis [for review see: Yang et al., 2017]. mGSK3 has also been suggested to be important for regulating oxidative stress (King et al., 2001), a function that it would share with non-mitochondrial GSK3 (Potz et al., 2018). Accordingly, lithium has been shown to have anti-oxidative effects by reducing mitochondrial dysfunction, increasing anti-oxidants, decreasing lipid peroxidation levels, and reversing complex I dysfunction (Macêdo et al., 2013; De Sousa et al., 2014; Scola et al., 2014; Kim et al., 2016).

### Autism spectrum disorder related pathways

Lithium and GSK3 also appear to affect different cell signaling mechanisms that have been associated with the development of subtypes of autism spectrum disorders (ASD). This may not be that surprising since bipolar disorder associated risk genes such as SHANK2 and ANK3 are shared between these disorders (Bi et al., 2012; Stahl et al., 2019; Zaslavsky et al., 2019; Kushima et al., 2022).

One interesting point of interaction involves the effect of lithium in attenuating symptoms of fragile X syndrome (Berry-Kravis et al., 2008; Yuskaitis et al., 2010; Liu et al., 2012; King and Jope, 2013; Liu and Smith, 2014). This disorder is classified as a subtype of ASD and is associated with a loss of function of the *FMR1* gene encoded on the human X chromosome. Lack of the encoded protein Fragile X messenger ribonucleoprotein 1 (FMRP; Herring et al., 2022) causes an array of cognitive, motor, mood, and anxiety symptoms (Arsenault et al., 2016; Hagerman et al., 2017). FMRP is a ribosome binding protein that has important roles in regulating mRNA functions (Oostra and Hoogeveen, 1997; Richter and Zhao, 2021). Several mRNA, including SHANK2, have been shown to be targets for FMRP (Dalal et al., 2021).

One consistent finding in fragile X syndrome is the increased activity of the mechanistic target of rapamycin (mTOR) signaling pathway (Sharma et al., 2010; Hoeffer et al., 2012). mTOR is itself involved in regulating mRNA translation and protein synthesis, as well as synaptic plasticity, cell growth, autophagy, and mitochondrial activity (Gingras et al., 2004; Hay and Sonenberg, 2004; Sarbassov et al., 2005; Morita et al., 2013). mTOR dysfunction is implicated in various disorders, including bipolar disorder where studies have found its activity to be decreased in brain and blood samples (Machado-Vieira et al., 2015; Vanderplow et al., 2021). Intriguingly, lithium treatment in a clinical trial and models of fragile X syndrome results in amelioration of behavioral abnormalities, decreased aberrant protein synthesis rates, and substantial decreases in mTOR activity (Berry-Kravis et al., 2008; Liu et al., 2012; Liu and Smith, 2014).

**Figure 2:**
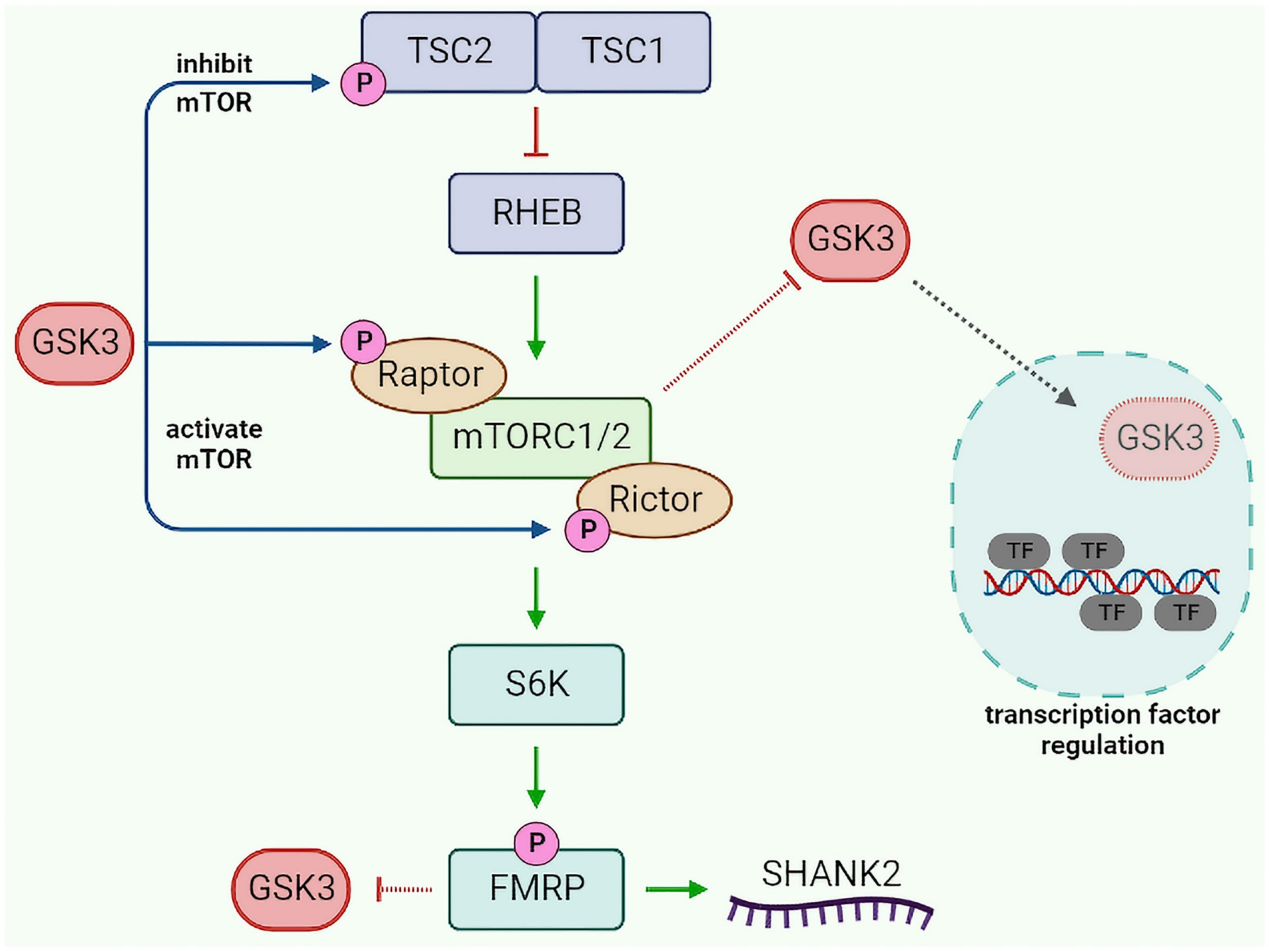
GSK3-mTOR-FMRP pathway crosstalk. GSK3 modulates mTOR activity by phosphorylating TSC2 (inhibit mTOR) or Raptor/Rictor (activate mTOR). Activated mTOR can inhibit GSK3 translocation to the nucleus, resulting in decreased GSK3-dependent regulation of transcription factors. Additionally, mTOR can increase FMRP activity (*via* S6K), which in turn can inhibit GSK3 and regulate SHANK2. GSK3, glycogen synthase kinase 3; TSC1/2, tuberous sclerosis 1/2; RHEB, ras homolog enriched in brain; mTORC1/2, mechanistic target of rapamycin complex 1/2; S6K, S6 kinase; FMRP, fragile X messenger ribonucleoprotein 1; SHANK2, SH3 and multiple ankyrin repeat domains protein 2; and TF, transcription factor.

Mechanistic target of rapamycin is an upstream regulator of FMRP. However, the translational regulation of FMRP causes indirect regulation of mTOR itself *via* a feedback loop (Bagni and Zukin, 2019). What is of high interest is the crosstalk between GSK3, mTOR, and FMRP (Figure 2). Evidence indicates that GSK3 *regulates* and *is regulated* by mTOR. GSK3 can either inhibit mTOR by phosphorylating its upstream effector tuberous sclerosis 2 (TSC2) or activate it by phosphorylating Raptor and Rictor and increasing their association to mTOR (Inoki et al., 2006; Stretton et al., 2015; Evangelisti et al., 2020). Alternatively, mTOR is capable of inhibiting GSK3 and limiting its nuclear translocation, thus constraining the activation/repression of transcription factor targets of GSK3 (Ka et al., 2014; Bautista et al., 2018; Evangelisti et al., 2020). Finally, lack of FMRP has been reported to activate GSK3 by reducing its inhibition by phosphorylation (Min et al., 2009). The intricacy of GSK3, mTOR, and FMRP signaling, as well as their shared lithium sensitivity and functional overlaps, suggest that this signaling module may play a role in the effects of lithium in both fragile X syndrome and bipolar disorder.

## Direct substrates of GSK3 regulated by lithium

While lithium can affect several biological functions, much less is known about the direct GSK3 substrates, by which it may mediate its therapeutic effects (Sutherland, 2011). It is important to note that GSK can act as a coincidence detector. Indeed, most GSK3 substrates need to first be primed (*via* phosphorylation) at a “priming sequence (in bold)”--Ser/Thr-X-X-X-**Ser/Thr-P**. GSK3 will recognize the primed Ser/Thr and further phosphorylate the substrate (Fiol et al., 1987). Phosphorylation by GSK3 often results in substrate inactivation or degradation *via* ubiquitination. While casein kinase II (CKII) was originally identified as a priming kinase for GSK3 substrates (Fiol et al., 1987), several other kinases have been shown to exert this function (Fiol et al., 1994; Del'Guidice et al., 2015). Interestingly, some priming kinases like PKA and ERK1/2 are regulated by extracellular signals, which support a role for GSK3 as a “coincidence detector” phosphorylating some of its substrates only under specific conditions. Consequently, inhibition of GSK3 may not result into specific biological outcomes if substrates are not primed to be modulated by GSK3 (Del’Guidice et al., 2015). We have already presented a few substrates including CLOCK, BMAL1, Raptor, and Rictor. In the remaining section of this review, the focus will be on a handful of substrates for which there is evidence of possible effects on bipolar disorder symptoms.

### cAMP response element-binding protein

cAMP response element-binding protein is one of the most characterized substrates of GSK3. It is a transcription factor that regulates an array of cellular activity including proliferation, growth, survival, differentiation, and inflammatory responses (Gonzalez and Montminy, 1989; Wen et al., 2010). Importantly, CREB regulates brain derived neurotrophic factor (BDNF) at promoter IV, and CREB is in turn activated *via* BDNF–TrkB signaling (Esvald et al., 2020). Thus, activating CREB leads to increased growth and plasticity. CREB is activated by phosphorylation on Ser133 by PKA, which is also used as a priming site for subsequent phosphorylation of Ser129 by GSK3, inactivating CREB (Gonzalez and Montminy, 1989; Fiol et al., 1994).

There are two single-nucleotide polymorphisms (SNPs) in the CREB1 gene that are associated with increased risk of bipolar disorder. These SNPs result in decreased expression and activity of both CREB protein and mRNA (Ren et al., 2014; Xiao et al., 2018). Analysis of protein interaction networks indicate that CREB can interact with several proteins encoded by GWAS-identified psychiatric disorder risk genes (Xiao et al., 2018). Furthermore, chronic lithium treatment increases CREB Ser133 phosphorylation and BDNF levels, preventing cognitive impairments and unregulated stress response (Grimes and Jope, 2001; Kopnisky et al., 2003; Miller et al., 2007; Fan et al., 2015; Rana et al., 2022).

Regulation of CREB activity by GSK3 may contribute to the regulation of several lithium responsive biological functions. For example, disrupted CREB activation results in highly fragmented bouts of activity during the night phase, elevated daytime activity, and disruption in cellular clock phase synchrony (Wheaton et al., 2018). This suggests an implication of the CREB pathway in regulating the circadian clock. However, the nature of the interaction between the effects of CREB, GSK3, and lithium to circadian regulation has not been characterized.

Another possible contribution of CREB to the effects of lithium and GSK3 inhibition can involve the regulation of Hebbian synaptic plasticity. Indeed, mice with loss of CREB function display impaired LTP and memory formation (Bourtchuladze et al., 1994; Kida, 2012). It is thus possible that increased GSK3 activity would inhibits CREB (leading to impaired LTP and strengthened LTD), whereas inactivation of GSK3 would restore CREB activity and induces LTP. Paradoxically, studies of chronic lithium treatment impact on CREB found decreased pSer133 CREB, as well as increased GluA1 internalization and decreased memory consolidation (Chen et al., 1999; Tardito et al., 2007; Mavrikaki et al., 2014; Amiri et al., 2020). However, lithium can also increase CREB activity (by increasing pSer133; Grimes and Jope, 2001; Kopnisky et al., 2003; Miller et al., 2007; Fan et al., 2015; Rana et al., 2022). This suggests that lithium has both inhibitory and disinhibitory effects on CREB in different neuronal populations or experimental systems.

Another important function of CREB is the regulation of anti-inflammatory pathways. Activated GSK3β causes decreased CREB translocation to the nucleus, resulting in decreased transcription of anti-inflammatory cytokines such as IL-10 (Platzer et al., 1999; Grimes and Jope, 2001; Wen et al., 2010). Inhibiting GSK3β has also been reported to modulate the activity of CREB to decrease the expression of pro-inflammatory cytokines IL-1β and TNFα (Song et al., 2003; Avni et al., 2010; Maixner and Weng, 2013). In addition, CREB can directly inhibit nuclear factor kappa-light-chain-enhancer of activated B cells (NF-κB) and prevent apoptotic and pro-inflammatory signals (Ollivier et al., 1996; Parry and Mackman, 1997). Taken together these observations suggest several mechanisms by which lithium could exert its anti-inflammatory actions *via* a modulation of GSK3 and CREB activity.

### Kinesins

Kinesins are a superfamily of motor enzymes. These proteins use energy from ATP hydrolysis to produce mechanical force and movement along microtubule tracks. There are several members of this family involved in a variety of functions including microtubule dynamics, cargo transport, chromosome segregation, cellular shape regulation, and mechanical integrity (Vale et al., 1986; Sheetz, 1987; Schroer et al., 1988; Ali and Yang, 2020). Kinesins are formed by combining two kinesin heavy chains and two light chains and are essential for anterograde axonal transport and the delivery of cargos to dendrites (Karasmanis et al., 2018; Ali and Yang, 2020). Kinesin’s light chain (KLC) and heavy chain (KHC) domains are phosphorylated by CKII, priming it for GSK3 phosphorylation (Morfini et al., 2002).

Phosphorylation of kinesins subunits on different sites by GSK3 can lead to different outcomes. For example, GSK3β phosphorylation of KIF1A at S402 impairs transport but does not regulate mobility, whereas phosphorylation of kinesin-1 motor domain at S314 halts motility without detaching from microtubules (Gan et al., 2020; Banerjee et al., 2021). In SH-SY5Y cells and neuronal hippocampal cultures, GSK3 phosphorylation of KLC1 and KLC2 results in decreased association of cargoes from the kinesin cargo system, indicating failed delivery of cargos to their target locations (Du et al., 2010; Morel et al., 2012). GSK3β also disrupts axonal transport by blocking translocation on microtubules, and this disruption is enhanced if kinesin-1 is reduced (Dolma et al., 2014). Interestingly, excessive GSK3β activity decreases the anterograde and retrograde segmental velocities of mitochondria, suggesting interrupted mitochondrial transport to dendrites and axons (Dolma et al., 2014).

Kinesin and GSK3 also play an important role in the trafficking of AMPA receptor subunits (Morfini et al., 2002; Setou et al., 2002; Nakata and Hirokawa, 2003; Du et al., 2010; Hirokawa et al., 2010). Specifically, phosphorylation of KLC2 by GSK3 results in a dissociation of membrane bound vesicles from kinesin, causing an internalization of AMPA-containing vesicles. Conversely, GSK3 inhibition results in increased surface GluA1 levels, which could be due to both less internalization and recycling inhibition (Du et al., 2010). This increased surface GluA1 was associated with improved acute mania-related and depression-related behaviors in mice, thus suggesting a possible therapeutic contribution (Du et al., 2010).

There is also evidence for a role of kinesin dysregulation in bipolar disorder. A post-mortem study has reported a reduction of kinesin-1 protein expression in the prefrontal cortex of subjects with bipolar disorder (Shao et al., 2016). Moreover, GSK3β inhibits neuronal transport by phosphorylating the kinesin light/heavy chains in an ANK3 deficiency model of bipolar disorder (Gottschalk et al., 2017). Decreased levels of heavy chain KIF5A and light chain KLC2, KLC3 and KLC4 in this model were rescued by lithium treatment. Lithium also rescued AMPA receptor availability and ameliorated bipolar disorder-related phenotypes in this same mouse model.

### Collapsin response mediator protein 2

The collapsin response mediator protein family contains five phosphoproteins (CRMP1-5). These proteins have important roles in axon formation, growth cone guidance, and cytoskeleton collapse *via* interactions with microtubules. CRMP2 is most studied out of the five proteins and seems to have additional roles other than regulating microtubule network, including axonal transport, vesicle trafficking, and neurotransmitter release (Gu and Ihara, 2000; Ip et al., 2014; Lee et al., 2019; Khanna et al., 2020). CRMP2 is phosphorylated by GSK3 at Thr509 and Thr514 sites (Cole et al., 2004; Yoshimura et al., 2005). GSK3-mediated phosphorylation of CRMP2 (pCRMP2) reduces polymerization of tubulin into microtubules, thus decreasing neurite outgrowth (Yoshimura et al., 2005). Non-phosphorylated CRMP2 promotes neurite outgrowth and microtubule stability. GSK3β has also been reported to modulate CRMP2 protein stability, as ablating GSK3β increased total levels of CRMP2 (Kim et al., 2019).

Dysregulations and increased phosphorylation of CRMP2 can result in dendritic spine defects or impaired microtubule dynamics, leading to smaller brain volume, which can contribute to pathology such as bipolar disorder (Crews et al., 2011; Zhang et al., 2016; Tobe et al., 2017). This is especially interesting as pCRMP2 is elevated in bipolar disorder stem cells, but only in lithium responsive patients (Tobe et al., 2017). In addition, increasing pCRMP2 or deleting the CRMP2 gene results in diminished mitochondrial trafficking, as well as alterations in mitochondrial morphology (Brustovetsky et al., 2021). Moreover, inhibiting CRMP2 phosphorylation reduces inflammatory response, *via* regulation of activated microglia and reactive astrocytes (Nagai et al., 2016).

Phosphorylation of CRMP2 can be regulated by lithium. Eliminating CRMP2 caused diminished spine density and loss of lithium responsiveness in neurons. Likewise, adding lithium decreased pCRMP2. These results were confirmed in human post-mortem brain samples and derived induced pluripotent cells that showed elevated pCRMP2 in bipolar disorder samples, which was normalized by lithium treatment (Tobe et al., 2017). Furthermore, increased pCRMP2 resulted in elevated soluble to polymerized tubulin ratio, which indicates enhanced microtubule dynamics. This increased ratio was also rescued by lithium treatment in cellular models of *ANK3* deficiency (Garza et al., 2018). Additionally, pCRMP2 suppressors mimicked the inhibitory effects of lithium in the amphetamine-induced-locomotor-hyperactivity model of mania, suggesting regulation of CRMP2 by dopamine receptors (Zhao et al., 2020). Interestingly, lithium causes a complete loss of pCRMP2 even in GSK3β ablated cells, suggesting that lithium targets GSK3α or other regulators of CRMP2 (Kim et al., 2019).

### Fragile X related protein 1

Fragile X related protein 1 (FXR1) is a member of the RNA binding protein family, which includes FMRP, FXR1, and Fragile X related protein 2 (FXR2). This family of proteins is involved in regulating mRNA stability, transport, splicing, and translation (Siomi et al., 1995). The Fragile X proteins are enriched in the brain, especially in neurons, with lower expression in glial cells (Tamanini, 1997; Zhang et al., 2014). Proteins from this family are known to interact with each other and often share similar target RNAs (Majumder et al., 2020; Taha et al., 2021). However, their functions are not always redundant (Winograd and Ceman, 2011). The functions of fragile X family proteins have been studied primarily in the context of fragile X syndrome and ASD. However, variants in the FXR1 locus have been genetically associated with bipolar disorder, schizophrenia, insomnia, and emotional regulation (Ripke et al., 2014; Liu et al., 2016; Bureau et al., 2017; Takata et al., 2017; Jansen et al., 2019). A study showed that FXR1 levels were decreased in a “post-traumatic stress disorder” mouse model (Nie et al., 2021). Moreover, regulation of FXR1 in mouse parvalbumin interneurons leads to the expression of schizophrenia-like behaviors (Shen et al., 2021).

Fragile X related protein 1 is primed by ERK1/2 phosphorylation, which then facilitates GSK3β phosphorylation and subsequent downregulation of FXR1, probably *via* ubiquitination (Del'Guidice et al., 2015; Qie et al., 2017). Targeting GSK3β or overexpressing FXR1 in the mouse prefrontal cortex led to behavioral effects similar to those of lithium and other mood stabilizers on anxiety-like behavioral dimensions in mice (Latapy et al., 2012; Del'Guidice et al., 2015; Khlghatyan and Beaulieu, 2020). Furthermore, human SNPs affecting the expression of *GSK3B* and *FXR1* genes in the human prefrontal cortex were shown to interact and affect emotional stability in controls, negative symptoms and antipsychotic drug responsiveness in schizophrenia, as well as symptom severity in bipolar disorder (Del'Guidice et al., 2015; Bureau et al., 2017; Rampino et al., 2021).

Chronic treatments with lithium, valproic acid, or lamotrigine increase FXR1 expression in the mouse striatum and prefrontal cortex. This increase is absent in β-arrestin 2 knockout mice, suggesting the βArr2/Akt/GSK3 pathway could be involved in regulating levels of FXR1 following these treatment (Del' Guidice and Beaulieu, 2015; Del'Guidice et al., 2015). Several biological functions that are relevant to the effects of lithium in bipolar disorder can potentially be affected by FXR1. For example, FXR1 has been shown to affect mitochondrial functions, oxidative stress, neurogenesis, and cell proliferation (Jagannathan et al., 2015; Patzlaff et al., 2017; Qie et al., 2017; Cao et al., 2019; Qi et al., 2020), which can be pertinent to phenotypes observed in studies of people with bipolar disorder and patient derived systems (Madison et al., 2015; Duong et al., 2021).

One major focus of recent studies has been the regulation of AMPA receptor transcription and surface expression by FXR1. Conditional knockout of FXR1 in excitatory neurons of the hippocampus exhibits increased protein synthesis-dependent LTP (Cook et al., 2014). FXR1 was shown to negatively regulate the translation of GluA2-5’UTR fluorescent protein reporters, thus potentially explaining this effect (Cook et al., 2014). Furthermore, somatic CRISPR/Cas9 GSK3β knockout or increased FXR1 expression led to decreased AMPA-mediated excitatory postsynaptic currents, and resulted in anxiolytic-like responses (Khlghatyan et al., 2018). Overexpression of FXR1 and GSK3β knockout also resulted in changes in the expression of pre-synaptic vGLUT1 and synaptic AMPA receptors subunits without affecting NMDA or GABA-A receptors (Khlghatyan et al., 2018). Together these observations indicate that GSK3β and FXR1 have multiple antagonistic effects in regulating basal AMPA-mediated excitatory neurotransmission and GluA1 and GluA2 surface expression, at least in the prefrontal cortex.

In addition to basal excitatory transmission, there is evidence for a role of GSK3β/FXR1 signaling in regulating homeostatic synaptic plasticity. FXR1 is downregulated during *in vitro* homeostatic scaling, or as a result of acute sleep deprivation, in the mouse prefrontal cortex (Khlghatyan et al., 2020). Downregulation of FXR1 was necessary for the homeostatic modulation of surface AMPA receptors under both conditions and preventing this downregulation during sleep deprivation altered electroencephalographic signatures. Interestingly, regulation of FXR1 in sleep deprivation and synaptic scaling was lithium sensitive and prevented by CRISPR/Cas9-mediated GSK3β inactivation (Khlghatyan et al., 2020).

Additional transcriptomic analysis using a ribotag reporter in the sleep deprivation model (Khlghatyan et al., 2020) revealed changes in the regulation of the Rho family GTPase Rac1, the Rho-associated serine/threonine kinase p21-activated kinase 3 (PAK3), and Microtubule-associated protein 2 (MAP 2). These proteins are involved in AMPA receptor trafficking and the regulation of LTP to LTD ratio. For example, PAK3 modulates GluA1 surface expression by phosphorylation, and supressing PAK3 activity results in altered AMPA receptor expression and impaired LTP (Boda, 2004; Hussain et al., 2015). Similarly, MAP 2 is involved in LTP response, and silencing MAP 2 blocks surface delivery of AMPA receptors (Fukunaga et al., 1996; Kim et al., 2020). This regulation of MAP 2 might work in conjunction with CRMP2 to stabilize microtubules and eventually affect trafficking *via* kinesins. Furthermore, various studies suggest that activated Rac1 is necessary for both AMPA receptor endocytosis during LTD, as well as increased surface AMPA receptors during LTP (Wiens, 2005; Xie et al., 2007; Benoist et al., 2013; Li et al., 2015; Guo et al., 2021).

Fragile X related protein 1 also regulates inflammatory responses by inhibiting the translation of TNFα mRNA, and knockdown of FXR1 removes the ability to inhibit LPS-induced TNFα protein production (Garnon et al., 2005; Khera et al., 2010). Moreover, Herman et al. (2018) found that FXR1 destabilizes pro-inflammatory transcripts. On the other hand, FXR1 could also increase pro-inflammatory responses in selected situations (Vasudevan and Steitz, 2007). Additionally, FXR1 has protective effects against mitochondrial oxidative stress and reactive oxygen species (ROS) production (Qi et al., 2020).

Unfortunately, no published studies have assessed the contribution of FXR1 to circadian rhythms, but *Fmr1; Fxr2* double knockout mice have arrhythmic activity, and either *Fmr1* or *Fxr2* knockout mice demonstrate shorter periods of free-running activity (Zhang et al., 2008). This suggests that FXR1 could potentially regulate circadian rhythms.

## Conclusion

**Figure 3:**
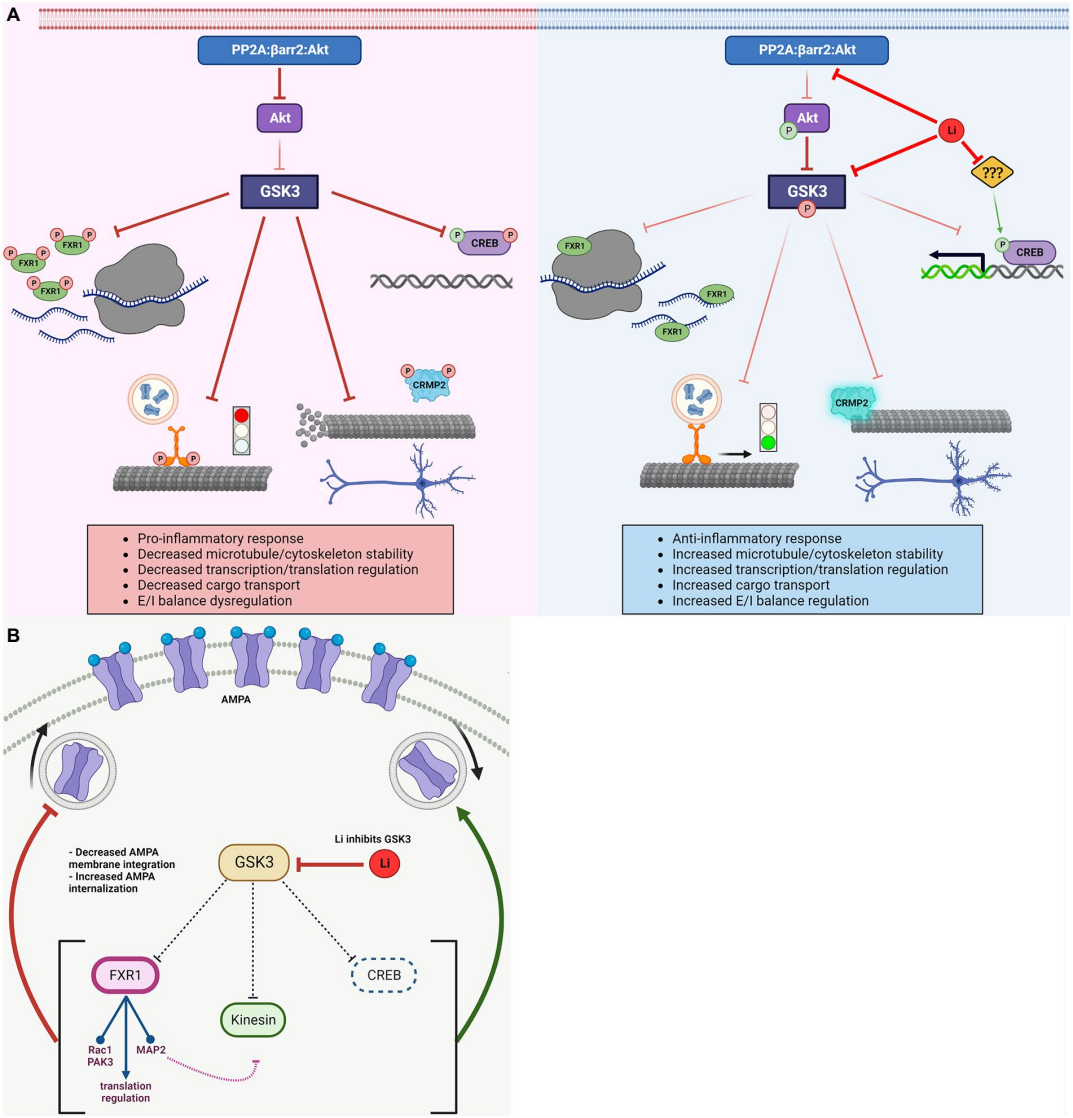
Regulation of GSK3 substrates and associated biological effects. **(A)** Activated GSK3 results in: decreased FXR1 mediated translational regulation, halted Kinesin and dissociated cargo, depolymerizing microtubule due to detached CRMP2, and inhibition of CREB-dependent transcription. Lithium inactivates GSK3 (directly and indirectly) resulting in: increased FXR1 mediated translation regulation, Kinesin mobility and delivery of cargo, CRMP2 association with (and increased stability of) microtubules, and activation of CREB-dependent transcription. Lithium has other targets, which may regulate CREB activity independently of GSK3. **(B)** GSK3 has various reported roles in regulating AMPA receptor trafficking and/or cell surface expression *via* modulation of different substrates. FXR1 *decreases* surface AMPA expression by regulating translational of AMPA receptor subunits mRNAs directly (mRNA, ribosomes) or indirectly by regulating expression of signaling molecules (Rac1, PAK3, and MAP2) involved in the regulation of receptor trafficking. CREB activity *increases* surface AMPA. Active kinesin also *increases* surface AMPA, but might be impeded by regulation of MAP2 and Rho GTPases by FXR1 (e.g., decreased MAP2 leads to unstable microtubules, preventing kinesin movement). Net result could depend on the relative activation/inhibition of specific pathways. GSK3, glycogen synthase kinase-3; FXR1, fragile X related protein 1; CRMP2, collapsin response mediator protein 2; CREB, cAMP response element-binding protein; Li, lithium; AMPA, α-amino-3-hydroxy-5-methyl-4-isoxazolepropionic acid; PAK3, p21-activated kinase 3; and MAP2, microtubule-associated protein 2.

The apparent non-pharmacological selectivity of lithium has impaired the development of better approaches for the management of bipolar disorder symptoms. Identification of GSK3 as a potential important target raised little hope since these kinases are involved in numerous biological functions. Furthermore, while some consequences of GSK3 inhibition correlates with biological outcomes of lithium treatment, this overlap has not been thoroughly characterized (Table 1). Identification of biological processes and related direct GSK3 substrates affected by bipolar disorder and lithium can provide a more mechanistic picture and point toward overlapping key biological functions mediating therapeutic effects (Figure 3A).

**Table 1:** Summary of GSK3 substrates and their effects on biological processes.

		Dopamine signaling and regulation	Excitatory signaling and transmission	Circadian rhythm	Inflammation	Mitochondria and oxidative stress
**CREB**	Role/Effect	–	Increases LTP and memory formation	Regulates function of circadian clock	Increases anti-inflammatory response	–
	Lithium vs. GSK3 inhibition	Not assessed with CREB	**Li:** both activates and inactivates CREB	Not assessed with CREB	**Li:** no direct results	Not assessed with CREB
			**GSK3-I:** activates CREB		**GSK3-I:** decreases pro-inflammatory cytokines *via* increased CREB activity	
	Correlation? (L vs. GI)	–	Partial	–	Yes (lithium, in general, decreases inflammatory response)	–
**Kinesin**	Role/Effect	–	Transports AMPA to dendrites for increased surface expression	–	–	Regulates mitochondrial transport to dendrites/axons
	Lithium vs. GSK3 inhibition	Not assessed with kinesin	**Li:** rescues levels of kinesin subunits and AMPA availability	Not assessed with kinesin	Not assessed with kinesin	Not assessed with kinesin
			**GSK3-I:** increases surface AMPA levels			
	Correlation? (L vs. GI)	–	Yes (above) and No (lithium can also causes decreased levels of AMPA surface expression)	–	–	–
**CRMP2**	Role/Effect	Decreases amphetamine-induced hyper-locomotion	Regulates vesicle trafficking and neurotransmitter release	–	Decreases inflammatory response	Regulates mitochondrial trafficking and morphology
	Lithium vs. GSK3 inhibition	Not assessed with CRMP2	Not assessed with CRMP2	Not assessed with CRMP2	Not assessed with CRMP2	Not assessed with CRMP2
	Correlation? (Li vs. GSK3-I)	**Li and GSK3-I:** decrease amphetamine-induced hyper-locomotion	–	–	Unknown (but lithium decreases inflammatory responses)	–
**FXR1**	Role/Effect	Is potentially regulated by the dopamine system for anxiolytic effect	Decreases AMPA surface expression and translation	FMR1 affects circadian functions. FXR1 not tested	Destabilizes and inhibits pro-inflammatory cytokines (especially TNFα)	Protects against oxidative stress and ROS production
	Lithium vs. GSK3 inhibition	**Li:** increases FXR1 only when βarrestin2 is present	**Li:** Increase FXR1 expression, impact on AMPA not tested in the context of lithium treatment	Not tested	Not tested	Not tested
		**GSK3-I:** conditional GSK3b-KO increases FXR1 levels	**GSK3-I:** decreases AMPA-mediated currents and signaling			
	Correlation? (L vs. GI)	Yes	Yes	–	Unknown (but lithium decreases inflammatory responses)	Unknown (but lithium alleviates oxidative stress)

^*^Li = Lithium; GSK3-I = GSK3 inhibition.

For example, CREB, CRMP2, and FXR1 contribute to the regulation of glutamatergic and dopaminergic signaling, inflammation, cellular morphology, microtubule stability, and mitochondrial functions (Figure 3). FXR1 may also be affected by mTOR signaling just as FMRP (Figure 2). Furthermore, kinesins contribute to the trafficking of glutamate receptors, mitochondria, and mRNAs implicated in local protein synthesis. Interestingly, these biological processes are often intertwined as cytokine signaling, mitochondrial regulation of inflammation, oxidative stress, and excitatory/inhibitory transmission balance have been shown to affect each other in multiple systems (Galic et al., 2012; Pandey et al., 2021).

However, divergences in the known contributions of some of these GSK3 substrates also exist. For instance, following inhibition of GSK3, FXR1 provides a block to increased AMPA cell surface expression while other substrates reported here enhance AMPA functions in response to lithium (Figure 3B). These effects could be dependent of experimental systems or may contribute to regulatory mechanisms involved in neuronal circuit associated to specific behavioral outcomes. A more systematic characterization of the impacts of GSK3 inhibition in relation to its various substrates may thus be needed to clarify contradictory observations and provide mechanistic basis for drug development.

Still, contradictions between the biological effects of lithium involving different GSK3 substrates may also point toward a more intriguing mechanism of action. One possibility is that several GSK3 substrates are affected by lithium to provide a homeostatic buffer, preventing extreme functional variation, and limiting maladaptive imbalance (Gray and Mcewen, 2013; Khlghatyan et al., 2020). In this perspective, GSK3 inhibition would enhance the function of substrates that oppose cellular plasticity in response to allostatic load. These various “breaks” would thus be working in concert to maintain brain functions within an optimal working equilibrium. Differential substrate priming may provide context-dependent flexibility for this action (Del’Guidice et al., 2015). Interestingly, this type of mechanism is compatible with the clinical effects of lithium as a mood stabilizer.

Finally, an important remaining frontier is the relationship between the various GSK3 substrates and the cellular mechanisms responsible for bipolar disorder. Is it that lithium reverses core changes induced by bipolar disorder? In which case, identifying key mechanisms would be simple! Alternatively, lithium may not be addressing causative mechanisms. It may just remediate symptoms by overriding the effects of bipolar disorder on brain functions. This latter scenario may be the most probable since GSK3 enzymes are also inhibited by several other drugs used in psychiatry, including antipsychotics and ketamine (Beaulieu et al., 2009). Identification of a link between GSK3 substrates and the effects of lithium on bipolar disorder symptoms could at best provide a better understanding of disease mechanisms, or at least better drug targets and significant biomarkers of treatment efficacy.

## Author contributions

JB and DC conducted research and wrote the manuscript. All authors contributed to the article and approved the submitted version.

## Funding

JB holds a Canada Research Chair (Tier1) in Molecular Psychiatry. DC is supported by an Ontario Graduate Scholarship. This work was also supported by a Project Grant from the Canada Institutes of Health Research (CIHR) Grant#: PJT-148568.

## Conflict of interest

The authors declare that the research was conducted in the absence of any commercial or financial relationships that could be construed as a potential conflict of interest.

## Publisher’s note

All claims expressed in this article are solely those of the authors and do not necessarily represent those of their affiliated organizations, or those of the publisher, the editors and the reviewers. Any product that may be evaluated in this article, or claim that may be made by its manufacturer, is not guaranteed or endorsed by the publisher.
